# A Second Look at Refractory Edema: Delayed Diagnosis of Paraneoplastic Cushing’s Syndrome in Small Cell Lung Cancer

**DOI:** 10.7759/cureus.99533

**Published:** 2025-12-18

**Authors:** George K Annan, Abigail Mills-Annoh, Donovan Persad, Obinna Orji, Issahaku Osmanu

**Affiliations:** 1 Internal Medicine, Piedmont Athens Regional Medical Center, Athens, USA

**Keywords:** cognitive bias, diagnostic delay, diagnostic error, ectopic acth production, ectopic acth syndrome (eas), extensive stage small cell lung cancer, fluid retention, heart failure, paraneoplastic cushing's syndrome, small-cell lung carcinoma

## Abstract

Paraneoplastic Cushing syndrome (PCS) is a rare manifestation of ectopic adrenocorticotropic hormone (ACTH) production, mostly associated with bronchial carcinoid and small cell lung cancer (SCLC). Its clinical manifestations: refractory hypertension, profound hypokalemia, metabolic alkalosis, worsening hyperglycemia, and edema, can easily be misattributed to more common conditions, especially in older adults with multiple comorbidities, leading to diagnostic errors.

We present a case of an 84-year-old man with a history of stage IA non-SCLC treated one year earlier, who developed progressive dyspnea, orthopnea, bilateral extremity edema, severe hypokalemia, metabolic alkalosis, and new-onset hypertension. His symptoms were initially managed as volume overload and diuretic-resistant heart failure in the outpatient setting. During hospitalization, persistent metabolic alkalosis, worsening hyperglycemia, resistant hypertension, and refractory hypokalemia prompted further evaluation. Laboratory studies demonstrated markedly elevated early morning cortisol (102.7 µg/dL) and ACTH (293 pg/mL). Computed tomography (CT) imaging revealed a new right infrahilar mass, extensive mediastinal adenopathy, and bilateral adrenal metastases. Endobronchial ultrasound-guided biopsy confirmed SCLC. The patient was diagnosed with paraneoplastic ACTH-dependent CS and initiated on systemic chemotherapy.

This case highlights several diagnostic vulnerabilities, including anchoring bias, confirmation bias, premature closure, and failure to integrate multiple abnormal findings into a unifying diagnosis. Earlier recognition of the characteristic cluster of hypercortisolism signs-refractory hypokalemia, metabolic alkalosis, resistant hypertension, and hyperglycemia- may have accelerated diagnosis and treatment. Clinicians should maintain a high index of suspicion for PCS in older adults with a history of lung cancer who present with unexplained electrolyte disturbances and rapidly worsening cardiometabolic parameters. Early diagnosis is critical given the high morbidity and mortality associated with untreated paraneoplastic Cushing’s syndrome.

## Introduction

Paraneoplastic ACTH-dependent Cushing syndrome (CS) is an uncommon but severe manifestation of ectopic adrenocorticotropic hormone production. Ectopic ACTH syndrome accounts for approximately 6-10% of all cases of endogenous CS [1]. This represents 10-20% of ACTH-dependent forms of Cushing syndrome, which themselves comprise 70-80% of all endogenous CS cases. Lung neuroendocrine tumors account for approximately 25% of cases, followed by small cell lung cancers (SCLC) (20%), with other sources being neuroendocrine tumors of the thymus, pancreas, and medullary thyroid carcinoma [2,3]. Patients typically present with symptoms related to underlying malignancy and rapid onset of severe hypercortisolism characterized by profound hypokalemia, metabolic alkalosis, hyperglycemia, and muscle weakness, often without the classic cushingoid features seen in other forms of CS [4,5].

These abnormalities are often initially attributed to more common conditions, including heart failure, diuretic use, thyroid disease, and worsening chronic diseases such as diabetes mellitus, especially in older adults with multimorbidity. This often leads to diagnostic errors. Diagnostic delays in paraneoplastic Cushing syndrome (PCS) are common and clinically meaningful. Hypercortisolism accelerates tumor progression, increases vulnerability to infection, worsens cardiometabolic dysfunction, and contributes to poor performance status, substantially limiting therapeutic options [6-8]. Prompt recognition requires clinicians to identify the hallmark constellation of metabolic disturbances and consider endocrine etiologies early.

We describe an older adult who presented with cough, dyspnea, edema, severe resistant hypertension, metabolic alkalosis, and electrolyte derangements that were initially attributed to volume overload and chronic lung disease. The diagnostic process ultimately led to the identification of extensive-stage SCLC, which caused ectopic ACTH production. We emphasize the diagnostic errors that contributed to the delayed recognition of this life-threatening syndrome.

## Case presentation

An 84-year-old man with a history of pre-diabetes, chronic obstructive pulmonary disease (COPD), a former smoker, and previously treated stage IA non-SCLC (left lower lobe, treated with Stereotactic Body Radiation Therapy) presented with cough, progressive shortness of breath, orthopnea, and bilateral lower extremity edema. Two weeks prior, outpatient clinicians treated his worsening edema and dyspnea with loop diuretics, and he was also started on nifedipine and losartan for hypertension.

In the emergency department, vital signs revealed blood pressure 216/98 mmHg, heart rate 104 beats/min, and respiratory rate 23 breaths/min. Physical examination demonstrated bilateral pedal edema extending to the mid-shins and bilateral upper extremity edema. Lung examination revealed no wheezing or crackles. The abdomen was obese but without palpable masses.

Initial laboratory evaluation showed mild thrombocytopenia (114 × 10^3^/µL), creatinine 1.10 mg/dL, potassium 2.9 mmol/L, bicarbonate 43 mmol/L, chloride 88 mmol/L, glucose 240 mg/dL, unremarkable liver function test, and elevated B-type natriuretic peptide (BNP) of 198 pg/mL. Arterial blood gas demonstrated pH 7.58 and PaCO₂ 42 mmHg, indicating primary metabolic alkalosis. Urinalysis was significant for glucosuria, otherwise unremarkable. Chest X-ray showed bibasilar atelectasis without evidence of pulmonary edema. He was admitted for decompensated heart failure. Pertinent admission laboratory findings are summarized in Table 1.

**Table 1:** Summary of relevant laboratory findings at presentation Metabolic alkalosis, renal potassium wasting, hyperglycemia, elevated cortisol, and ACTH suggested an ACTH-dependent Cushing's syndrome. BNPL: brain natriuretic peptide; ACTH: adrenocorticotropic hormone

Test	Result	Range
Hemoglobin	16.4 g/dL	13.8-17.2 g/dL
White cell count	9.7 × 10^3^/µL	4.0-10.50 × 10^3^/µL
Platelet	114 × 10^3^/µL	130-400 × 10^3^/µL
Sodium	142 mmol/L	133-145 mmol/L
Potassium	2.9 mmol/L	3.3-5.1 mmol/L
Chloride	88 mmol/L	98-108 mmol/L
Bicarbonate	43 mmol/L	22-32 mmol/L
Creatinine	1.10 mg/dL	0.50-1.20 mg/dL
BNP	198.8 pg/mL	10.0-100.0 pg/mL
Albumin	3.7 g/dL	3.0-5.0 g/dL
Glucose	240 mg/dL	70-100 mg/dL
Serum cortisol	102.7 µg/dL	6.7-22.6 µg/dL
Plasma ACTH	293 pg/mL	6-50 pg/mL
Urine chloride	73 mmol/L	
Urine potassium	38 mmol/L	

Despite diuresis with IV furosemide, he continued to demonstrate metabolic alkalosis and worsening hypokalemia (nadir 2.8 mmol/L), requiring repeated potassium supplementation. Hyperglycemia persisted with capillary blood glucose 170-300 mg/dL, requiring escalating insulin doses. Blood pressures remained elevated despite escalation of losartan and nifedipine. Echocardiogram on day 2 of admission was unremarkable with an ejection fraction of 55-60% and normal diastolic function. Doppler ultrasound of the lower and upper extremities did not reveal deep vein thrombosis.

**Figure 1:**
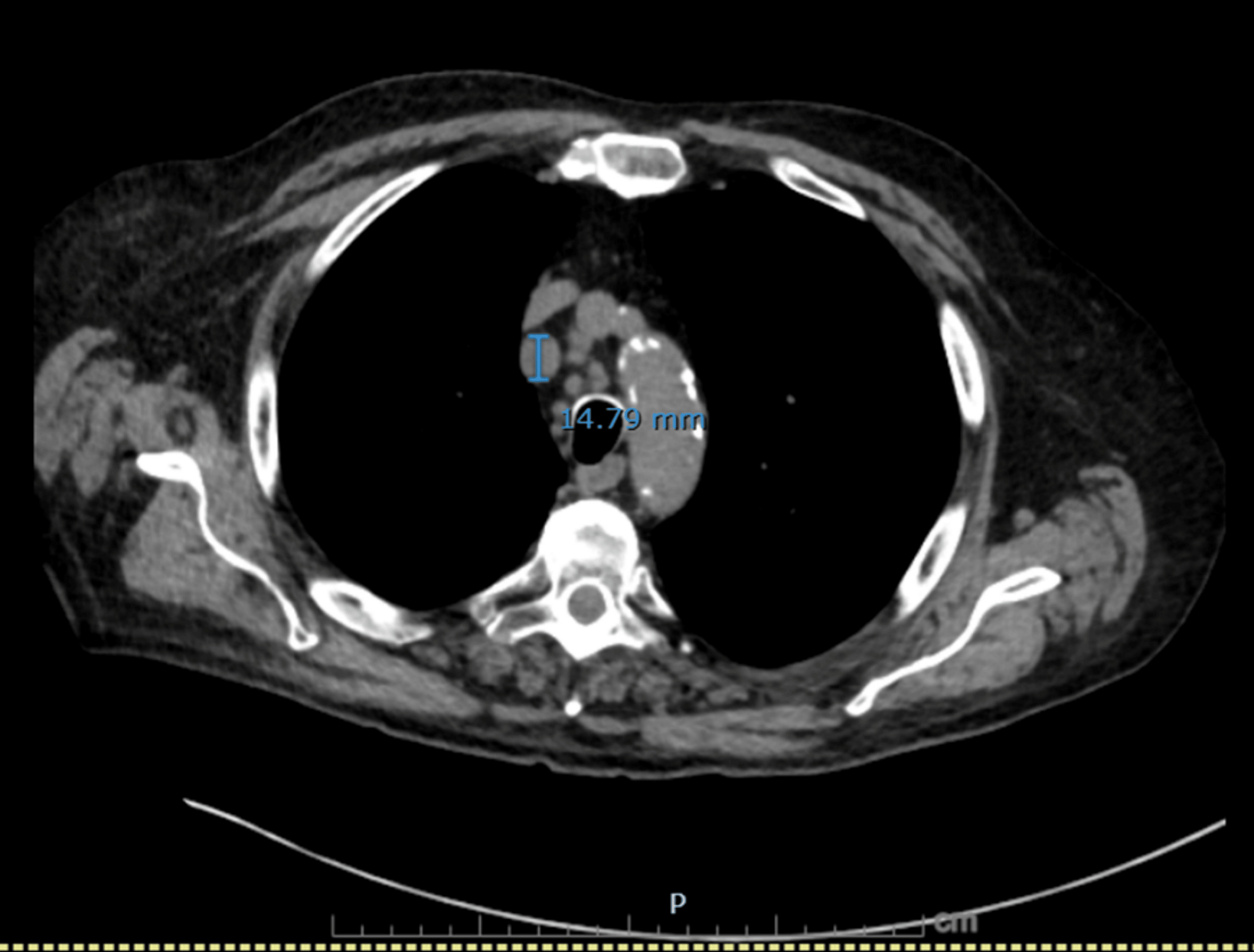
Axial CT chest showing an enlarged right paratracheal lymph node. Axial image demonstrates a right paratracheal lymph node measuring 14.8 mm in short axis, concerning for malignant nodal involvement.

**Figure 2:**
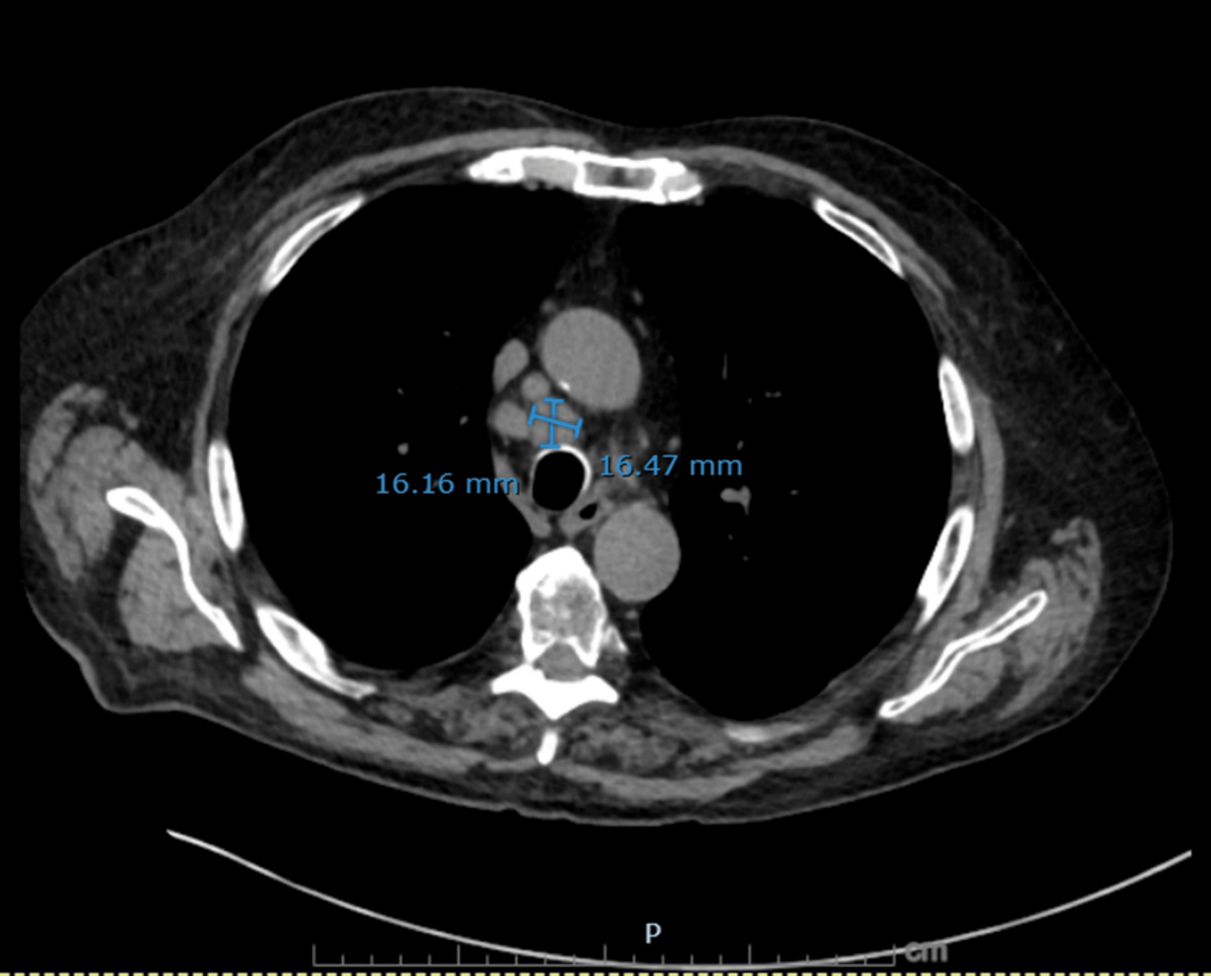
Non-contrast axial CT chest showing a dominant right paratracheal lymph node A right paratracheal lymph node measuring 16.2 × 16.5 mm is demonstrated, further supporting malignant mediastinal involvement in small cell lung cancer.

**Figure 3:**
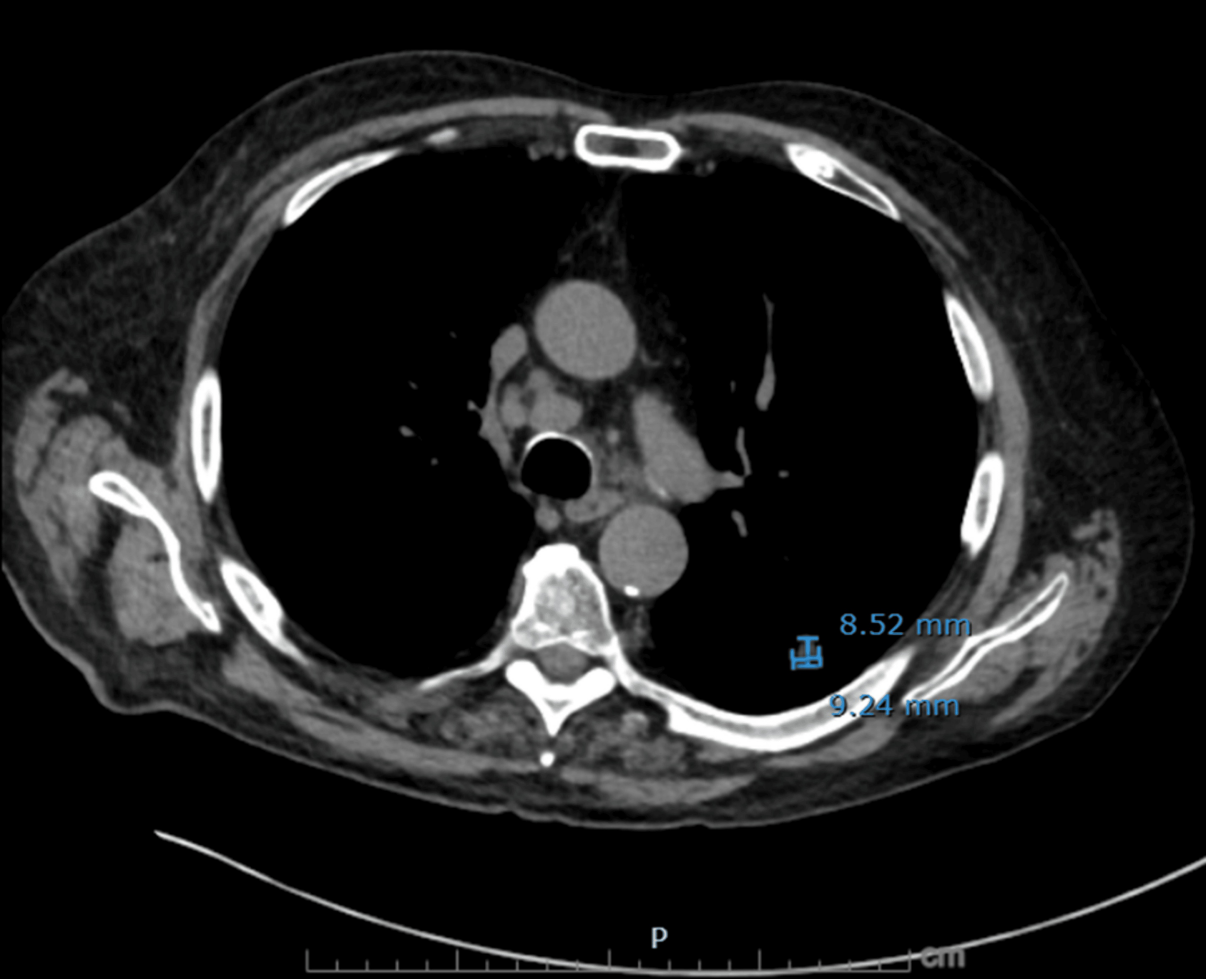
Axial non-contrast CT chest demonstrating residual treated left lower lobe lesion A spiculated nodule in the left lower lobe measuring 9.2 mm (AP) × 8.5 mm (transverse) on image 60, slightly decreased from the prior measurement of 9.3 × 10.6 mm, corresponding to the site of previously treated squamous cell carcinoma.

**Figure 4:**
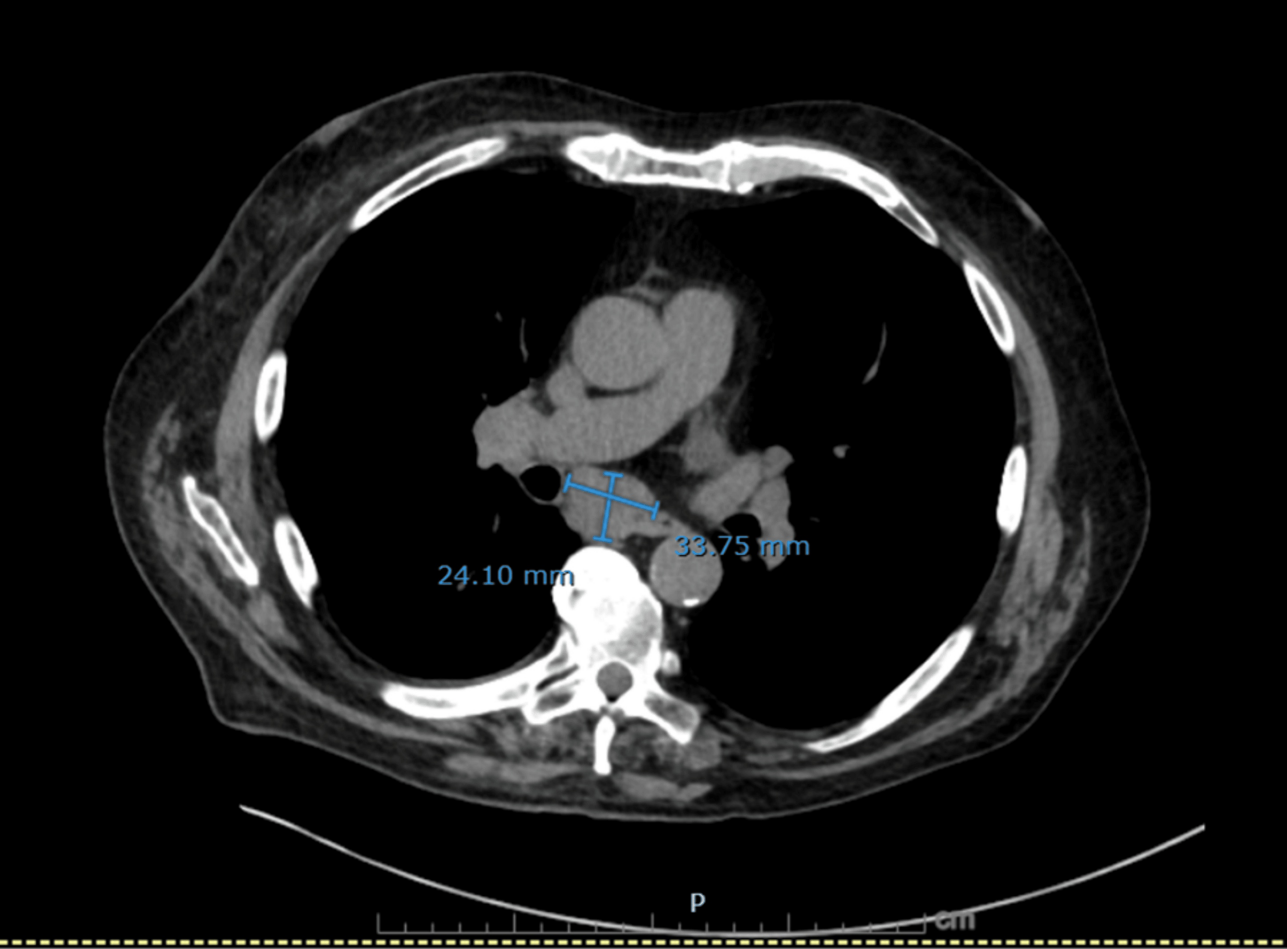
Axial non-contrast CT chest showing markedly enlarged subcarinal lymph node A dominant subcarinal lymph node measuring 24 × 34 mm, highly suspicious for malignant mediastinal involvement.

**Figure 5:**
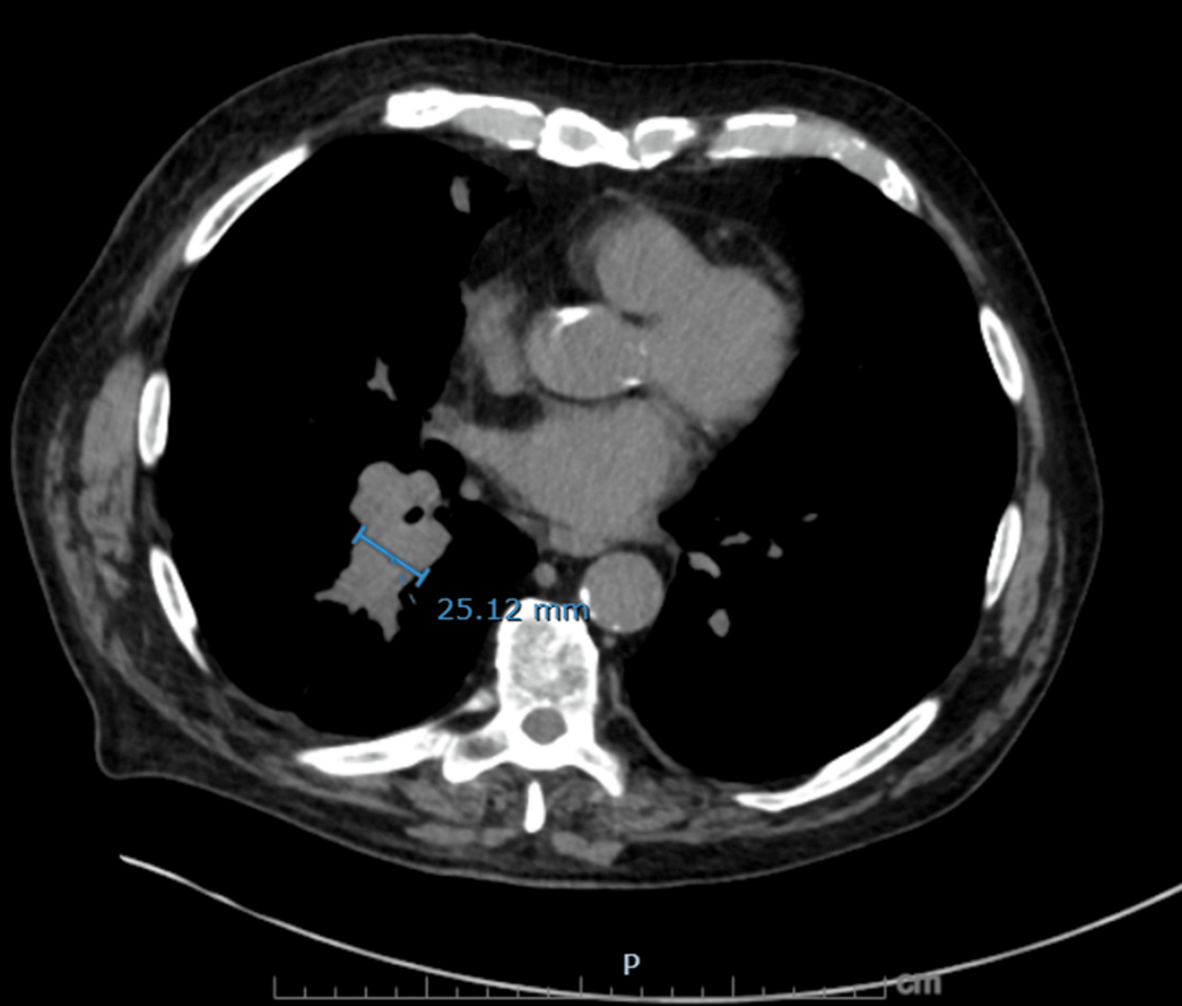
Axial non-contrast CT chest showing right infrahilar mass-like fullness Soft tissue density in the right lower lobe infrahilar region measuring up to 25 mm in transverse diameter, concerning for primary malignant involvement.

**Figure 6:**
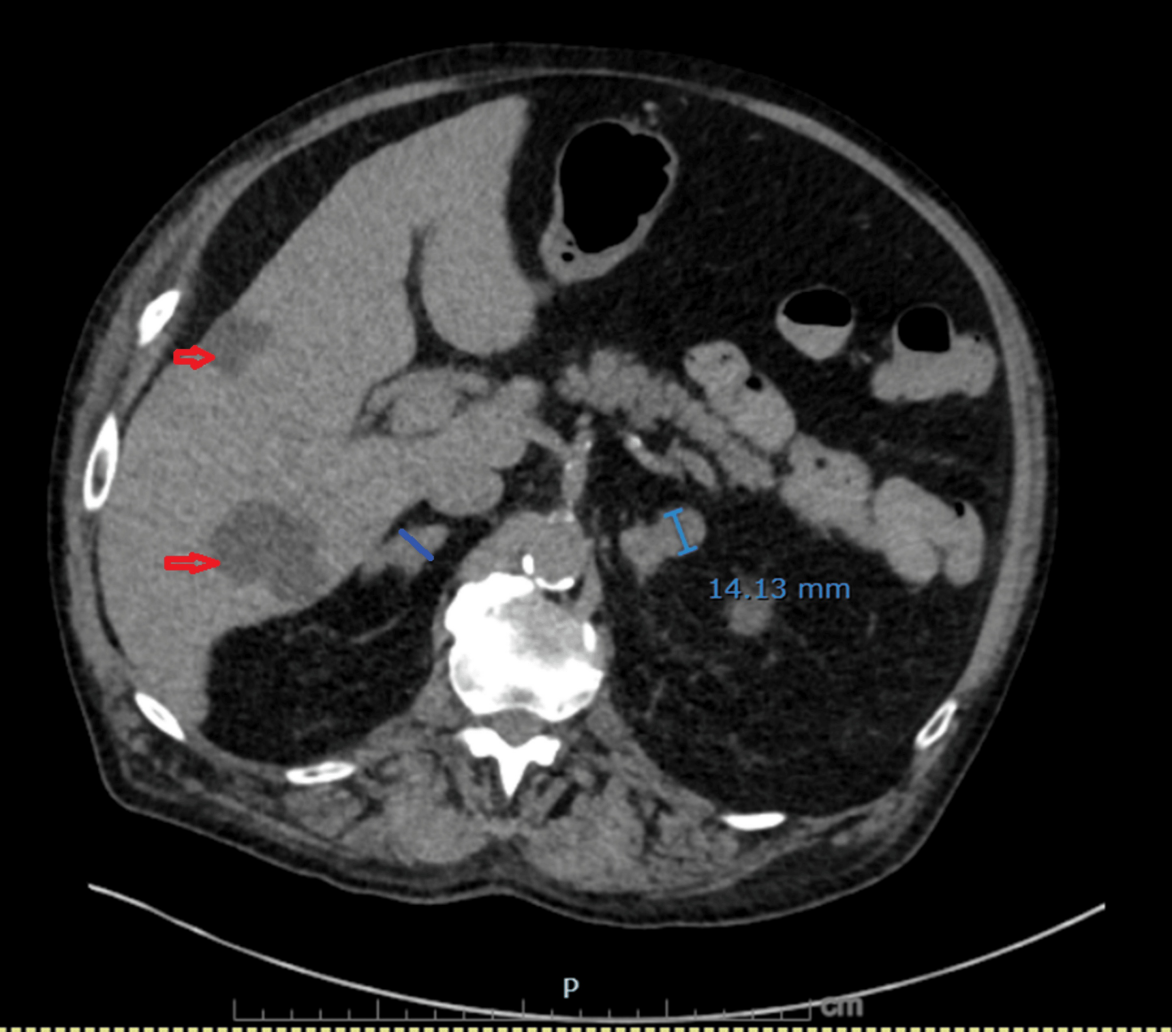
Non-contrast CT demonstrating bilateral adrenal metastases Nodular enlargement of both adrenal glands has progressed compared with prior imaging: the left adrenal lateral limb measures 14 mm (previously 9.2 mm) and the right adrenal body measures 12.4 mm (previously 7 mm). Multiple benign hepatic cysts are also visualized (red arrows).

On hospital day 3, diagnosis was reassessed, and differentials were broadened to include endocrine causes of hypertension with metabolic alkalosis. Urine electrolytes revealed high urine chloride (73 mmol/L) and potassium (38 mmol/L), suggestive of potassium wasting from possible mineralocorticoid excess. Subsequent testing revealed markedly elevated serum cortisol (102.7 µg/dL) and plasma ACTH (293 pg/mL), suggesting an ACTH-dependent process. Given his significant history of smoking and treated NSCLC, a CT chest/abdomen/pelvis was done, which showed a new right infrahilar mass, mediastinal lymphadenopathy, and nodular fullness of both adrenal glands concerning for metastatic disease (Figures 1-6).

Bronchoscopy with endobronchial ultrasound-guided transbronchial needle aspiration of the subcarinal (station 7) and right hilar (station 10R) lymph nodes revealed small cell carcinoma. He was diagnosed with extensive-stage SCLC with adrenal metastases and paraneoplastic ACTH-dependent Cushing syndrome. Systemic chemotherapy with carboplatin, etoposide, and atezolizumab was initiated.

## Discussion

PCS caused by ectopic ACTH secretion is associated with significantly higher morbidity and mortality than other forms of hypercortisolism. Patients experience universal acute complications and have markedly shortened survival, with median survival reported as low as 3-4 months in those with SCLC [7-9]. Early mortality is common, with most deaths occurring within weeks to months of diagnosis and frequently driven by opportunistic infections, thromboembolic events, and severe metabolic derangements [6,7]. Hypercortisolism itself impairs the ability to deliver effective cancer therapy, increasing the risk of treatment-related complications and reducing chemotherapy response rates [6]. Ectopic ACTH production is therefore considered the most lethal etiology of Cushing syndrome, with tumor progression and infection being the predominant causes of death.

Diagnostic error is the failure to establish an accurate and timely explanation of the patient's health problem(s) or communicate that explanation to the patient [10]. Diagnostic errors remain a significant contributor to patient harm, with estimates suggesting they affect 5-25% of patients [11,12]. These errors often arise not from knowledge deficits but from cognitive heuristics that clinicians rely on to navigate diagnostic uncertainty. While heuristics are essential for efficiency, they can predispose clinicians to systematic errors, especially when used uncritically or in complex cases [13,14]. Three cognitive pitfalls are particularly relevant in diagnostic error: anchoring bias (fixating early on a diagnosis and failing to adjust as new data emerge), premature closure (ceasing further diagnostic inquiry once an initial label is applied), and diagnostic momentum (the inertia created as more clinicians accept and act upon an early diagnostic impression) [15,16]. These processes can perpetuate incorrect diagnoses and delay definitive care.

For our patient, the initial clinical presentation of dyspnea, orthopnea, bilateral edema, and markedly elevated blood pressure in this older adult reasonably prompted consideration of several common cardiopulmonary and renal conditions. Acute decompensated heart failure was an early working diagnosis given his orthopnea, lower extremity edema, and elevated BNP. However, this diagnosis became less convincing as objective data accumulated. The patient had no pulmonary edema on chest imaging and preserved left ventricular systolic and diastolic function on echocardiography. Additionally, the severity of metabolic alkalosis and hypokalemia was disproportionate to the degree of diuretic exposure and volume status. These discrepancies argued against heart failure as a unifying diagnosis.

A COPD exacerbation was also considered due to the patient’s chronic lung disease and dyspnea. Yet he had no wheezing, no infectious symptoms, and no significant gas-exchange abnormality. His arterial blood gas (ABG) demonstrated metabolic alkalosis without primary respiratory acidosis. Moreover, his dyspnea improved early in the hospitalization, while the metabolic disturbances worsened, further making COPD a less likely diagnostic consideration. Renal causes of edema and hypertension, including nephrotic syndrome and intrinsic kidney disease, were evaluated. The patient had normal albumin and creatinine, and no significant proteinuria or hematuria on urinalysis, findings that could not explain his systemic edema. Similarly, acute or chronic kidney disease could not account for the combination of profound hypokalemia, metabolic alkalosis, and high urine chloride, which instead suggested an active mineralocorticoid process with renal wasting.

Primary hyperaldosteronism was a strong possibility, particularly given the combination of hypertension, hypokalemia, and metabolic alkalosis. However, the patient’s severe hyperglycemia, thrombocytopenia, new constitutional swelling of the upper extremities, and rapid symptom evolution were atypical for isolated hyperaldosteronism. Additionally, bilateral adrenal fullness seen on CT imaging was more consistent with adrenal metastases than with aldosterone-producing adenomas or hyperplasia. The degree of metabolic derangements also exceeded that typically observed in primary hyperaldosteronism, prompting evaluation for cortisol excess.

CS emerged as a unifying explanation for the multisystem abnormalities. The biochemical pattern, including severe metabolic alkalosis, renal potassium wasting, hyperglycemia, and resistant hypertension, is characteristic of activation of glucocorticoid and mineralocorticoid receptors. Markedly elevated cortisol and ACTH levels confirmed ACTH-dependent hypercortisolism. In older adults, pituitary Cushing disease typically evolves more slowly and is rarely associated with such profound hypokalemia [17,18]. Therefore, ectopic ACTH secretion became the leading diagnosis. The patient’s imaging, showing a new right infrahilar mass, progressive mediastinal lymphadenopathy, and bilateral adrenal enlargement, provided a clear source, later confirmed as extensive-stage SCLC.

This diagnostic trajectory illustrates how complex presentations can lead clinicians toward more common conditions, even when early clues point elsewhere. Several cognitive and system-level factors contributed to the delayed recognition of hypercortisolism. Anchoring on heart failure, a condition that fit parts of the patient’s presentation, discouraged re-examination of the initial differential when laboratory data did not fully align. Metabolic abnormalities were at first treated as isolated issues rather than components of a broader endocrine disorder. The patient’s prior non-SCLC had been in remission, which may have reduced the perceived likelihood of malignancy-related pathology, despite the well-known risk of second primary lung cancers and transformation events in older adults with smoking histories. Older adults with a history of smoking who have survived cancer face a substantially elevated risk of developing second primary lung cancers, with the risk persisting for decades after smoking cessation. Among lung cancer survivors, the 10-year cumulative incidence of a second primary lung cancer is approximately 8-15%, which is considerably higher than rates observed in general lung cancer screening populations [19,20].

The availability of more familiar explanations for dyspnea, edema, and hypertension, such as heart failure, may have overshadowed the classical biochemical signature of hypercortisolism. Recognition of ectopic ACTH production requires integrating disparate clinical findings into one physiological pathway. When evaluated collectively rather than individually, these abnormalities strongly suggest cortisol excess long before imaging or biopsy results are available.

Earlier consideration of endocrine etiologies could have expedited diagnosis, reduced unnecessary diuresis, and allowed earlier initiation of appropriate oncologic therapy. PCS from SCLC is associated with rapid clinical decline, impaired immunity, and decreased tolerance to chemotherapy. Prompt recognition may therefore improve both morbidity and the feasibility of cancer-directed treatment. This case reinforces the importance of revisiting and broadening the differential diagnoses when expected clinical improvement does not occur, particularly in older adults with prior malignancy and new multisystem derangements. Incorporating metacognitive strategies, actively questioning initial assumptions, seeking disconfirming evidence, and engaging in reflective practice can mitigate such errors [13].

## Conclusions

This case emphasizes the importance of considering paraneoplastic ACTH-dependent CS in older adults presenting with unexplained hypokalemia, metabolic alkalosis, hyperglycemia, and resistant hypertension, particularly in patients with a history of lung cancer. Diagnostic error arose from anchoring on cardiopulmonary etiologies and failure to synthesize metabolic abnormalities into a unifying diagnosis. Early recognition of hypercortisolism is essential, as untreated ectopic ACTH production rapidly worsens morbidity and limits therapeutic efficacy in SCLC.
